# Supplementary material for: The ion channel function of polycystin‐1 in the polycystin‐1/polycystin‐2 complex
Source: EMBO Rep. 2019 Aug 22;20(11):e48336. doi: 10.15252/embr.201948336 (PMC6832002; doi:10.15252/embr.201948336)
Supplement: Supplementary file 1 — Appendix [file EMBR-20-e48336-s001.pdf]

Appendix Supplementary Information

The ion channel function of the polycystin-1 in the polycystin-1/polycystin-2 complex

Zhifei Wang, *et al.*

Appendix Figure S1…..…………………………………….P2

Appendix Figure S2 ………………………………………..P3

Appendix Figure S3 ………………………………………..P4

Appendix Figure S4 ………………………………………..P5

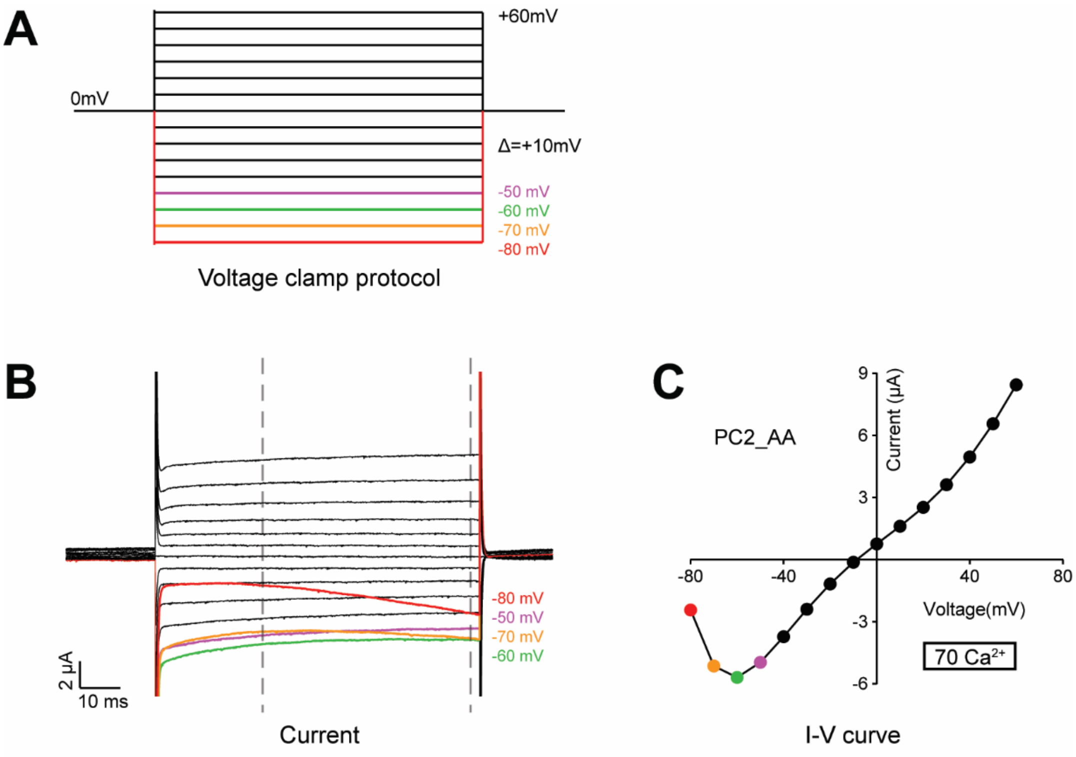

Appendix Figure S1 - The characteristic current of PC2_AA mutant in a bath solution containing 70 mM Ca^{2+}, showing how the trough of the I-V curve in strong negative voltage is generated.

A. A typical voltage clamp protocol used for recording. The first several tested voltages, from -80 mV to -50 mV, were marked in colors.

B. The corresponding currents of the PC2_AA channel. Colored traces indicated the currents at indicated voltages. Current at the first negative voltage application (-80 mV) is significantly smaller than that in the following several voltages. This result may be caused by the time delay between the start of the Ca^{2+} influx and the activation of Ca-induced chloride channel. The portions of currents between the dashed lines were averaged for generating the I-V curve.

C. I-V curve generated from the recording result. Thus, the channel seems to be slowly activated by hyperpolarizations, and data at -40 to -70 mV were acquired when channel is more activated than at -80mV. To avoid it, we tried to record with longer protocol to increase holding time at -80 mV. However, it only led to slow development of larger currents (may be due to the increasing of the Cl^- channels activated by Ca^{2+} influx) without changing the final shape of I-V curve.

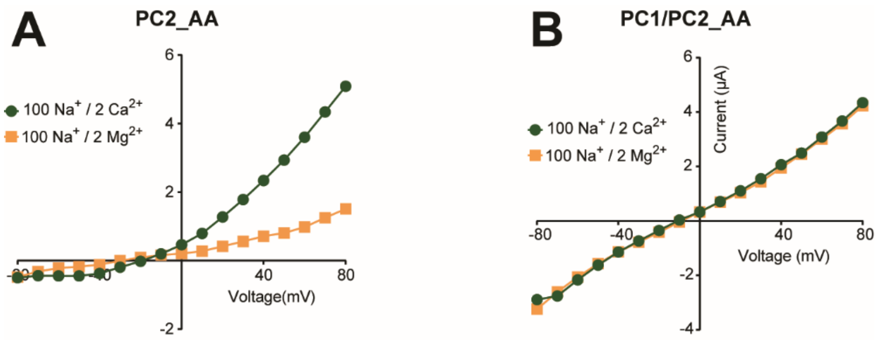

Appendix Figure S2 - Mg2+ inhibition of the PC2_AA and PC1/PC2_AA channels.

A, B. Representative I-V curves of PC2_AA (A) and PC1/PC2_AA (B) channels in bath solutions containing the indicated ions (in mM), showing both 2 mM Ca^{2+} and 2 mM Mg^{2+} inhibit the inward current of PC2_AA channel, but not that of PC1/PC2_AA channel. Also, 2 mM Mg^{2+} inhibits more on the outward current of PC2_AA than 2 mM Ca^{2+}.

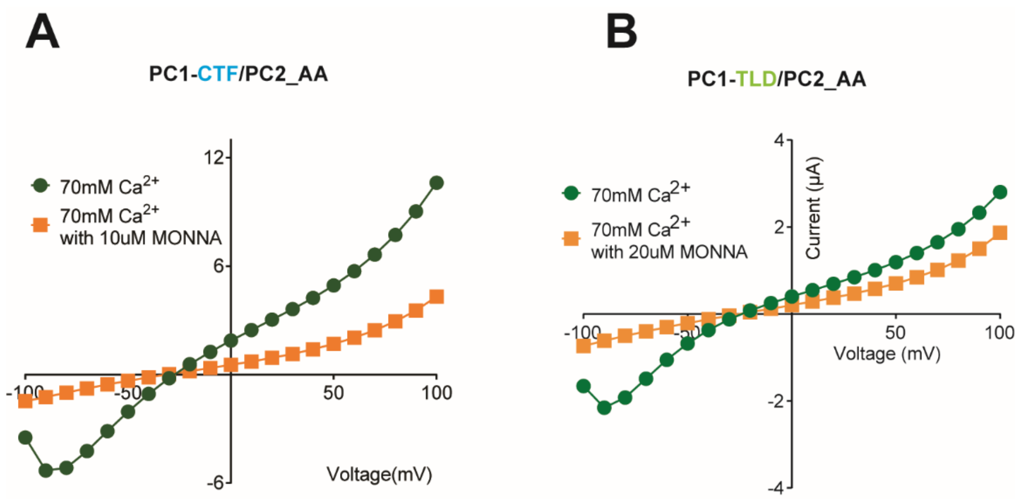

Appendix Figure S3 - MONNA inhibits the current of PC1-CTF/PC2_AA PC1-TLD/PC2_AA in 70 mM Ca^{2+}.

A, B Currents of the PC1-CTF/PC2_AA (A) and PC1-TLD/PC2_AA (B) channel in 70 mM Ca^{2+} in the presence or absence of MONNA with indicated concentrations.

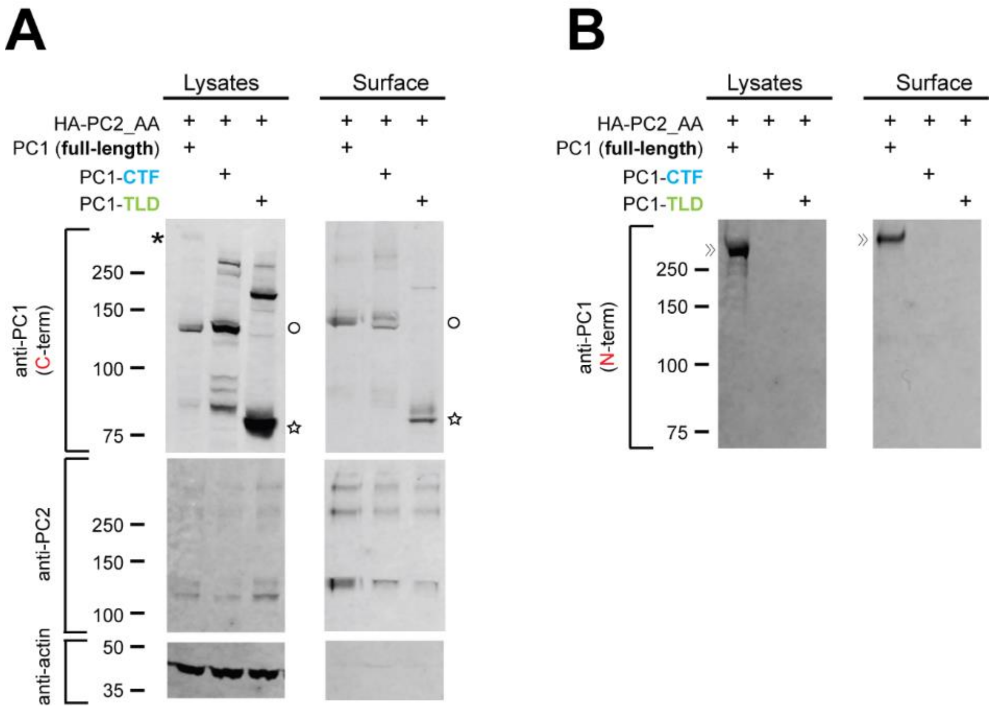

Appendix Figure S4 - The western blot of oocyte lysate and surface samples showing the surface expression of complexes formed by PC2 with either full-length PC1, PC1-CTF, or PC1-TLD.

A. Images the same western blot images shown in Fig. 4J. Bands of full-length (asterisk), GPS-cleaved CTF or expressed CTF fragment (open circle), and TLD (star) of PC1 are indicated.
B. The samples were also blotted with the anti-PC1 N-terminus antibody 7e12. Only one band, which is in the full-length PC1 samples (in both lysate and surface samples), was detected. We believe the band is the cleaved NTF (arrow head). Full-length PC1 band may be too weak to be seen.
