# Supplementary material for: The ion channel function of polycystin‐1 in the polycystin‐1/polycystin‐2 complex
Source: EMBO Rep. 2019 Aug 22;20(11):e48336. doi: 10.15252/embr.201948336 (PMC6832002; doi:10.15252/embr.201948336)
Supplement: Supplementary file 2 — Expanded View Figures PDF [file EMBR-20-e48336-s002.pdf]

Expanded View Figures

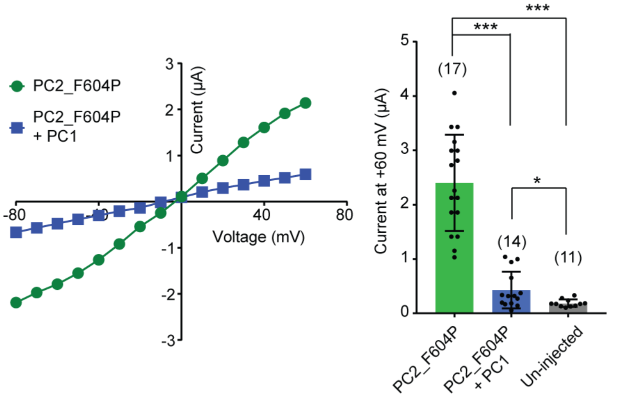

Figure EV1. Coexpression of PC1 greatly inhibited the current of PC2_F604P.

Left: representative current–voltage (I–V) curves of the indicated protein combinations. Right: Scatter plot and bar graph show the average currents at +60 mV. Oocyte numbers are indicated in parentheses. Data are presented as mean ± SD in bar graph (*$P < 0.05$, ***$P < 0.001$, Student's *t*-test).

Source data are available online for this figure.

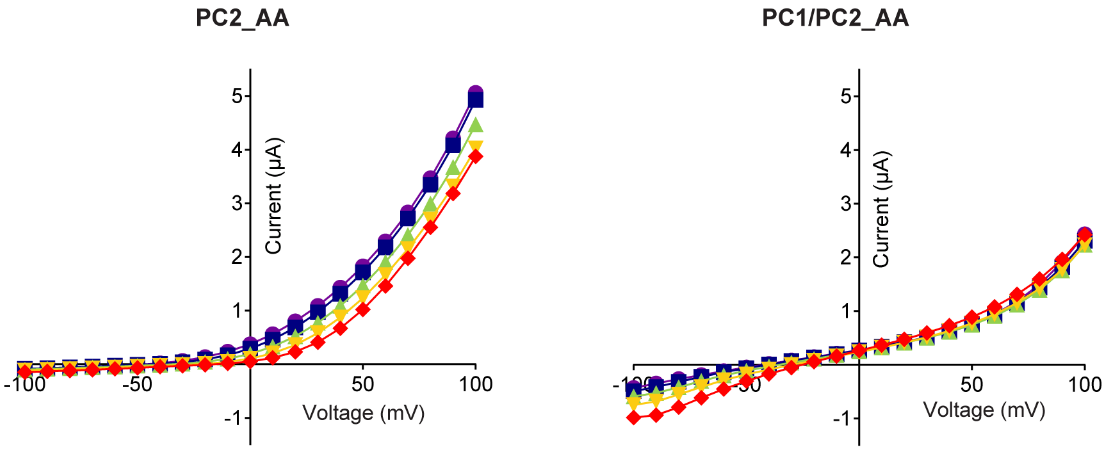

	[Ca²⁺] (mM)	[NMDG⁺] (mM)	[MONNA] (μM)	HEPES (mM)	pH
●	2	100	20	2	7.5
■	5	95	20	2	7.5
▲	10	90	20	2	7.5
▼	25	67.5	20	2	7.5
◆	70	0	20	2	7.5

Figure EV2. Representative I–V curves of PC2_AA (left) and PC1/PC2_AA (right) in solutions with varying Ca^{2+} concentration.

Components of these solutions are shown in the table. 20 μM of MONNA was included in all solutions, and the osmolarity was compensated by adding corresponding concentrations of $NMDG^+$, which is not permeable through these channels.

© 2019 The Authors

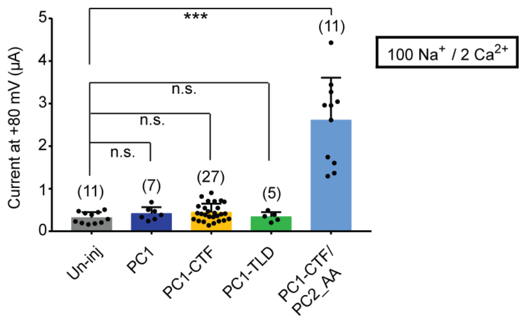

Figure EV3. Expressing full-length PC1, PC1-CTF, or PC1-TLD without PC2_AA in *Xenopus* oocytes did not give rise to significant current.

Scatter plot and bar graph show the average current sizes of oocytes expressing indicated full-length or fragment PC1 proteins in a solution containing 100 mM Na^+ and 2 mM Ca^{2+}. Oocytes expressing both PC1-CTF and PC2_AA were used as a positive control. Oocyte numbers are indicated in parentheses. Data are presented as mean ± SD in bar graph (n.s.: not significant, $***P < 0.001$, Student's *t*-test).

Source data are available online for this figure.

 © 2019 The Authors

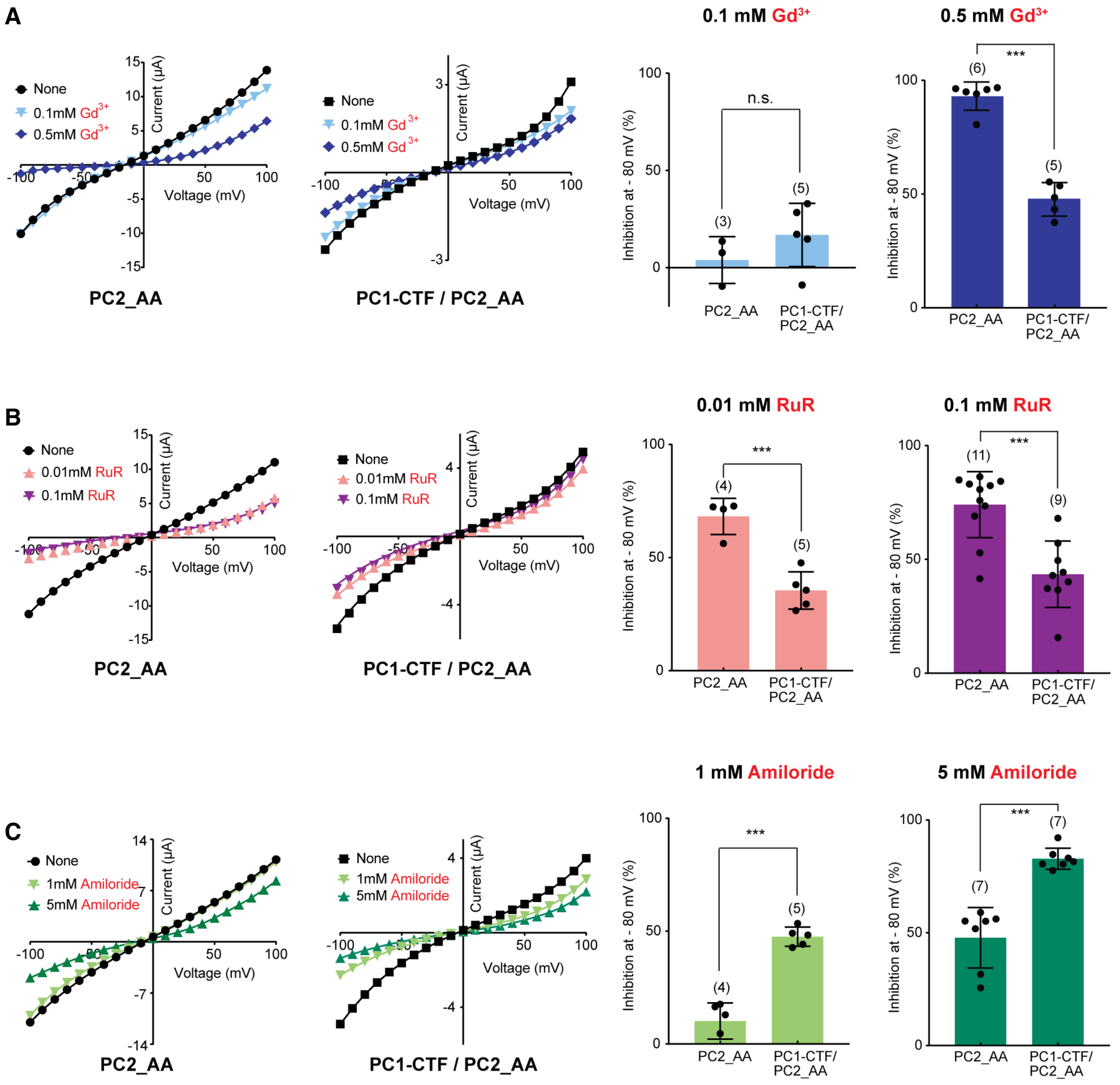

Figure EV4. The different effects of three channel blockers on the currents of the PC2_AA and the PC1-CTF/PC2_AA channels.

A–C Left two charts: representative I–V curves of the indicated two channels in the bath solution containing 100 mM Na^+ in the absence and presence of the indicated three blockers with two concentrations: 0.1 and 0.5 mM Gd^{3+} (A), 0.01 and 0.1 mM ruthenium red (RuR) (B), and 1 and 5 mM Amiloride (C). Right: Scatter and bar graphs show the inhibition (%) of the channel currents at −80 mV caused by corresponding blockers in two different concentrations, as shown on the left. Oocyte numbers are indicated in parentheses. Data are presented as mean ± SD in bar graph (n.s.: not significant, ***$P < 0.001$, Student's *t*-test).

Source data are available online for this figure.

Figure EV5. Pore mutations change the ion permeability of the PC1/PC2_AA channel.

Representative I–V curves show the shifts of reversal potential caused by indicated mutations in bath solutions containing 100 mM of indicated ions. Compared to group 2 mutations, group 1 mutations cause more dramatic reversal potential shifts.

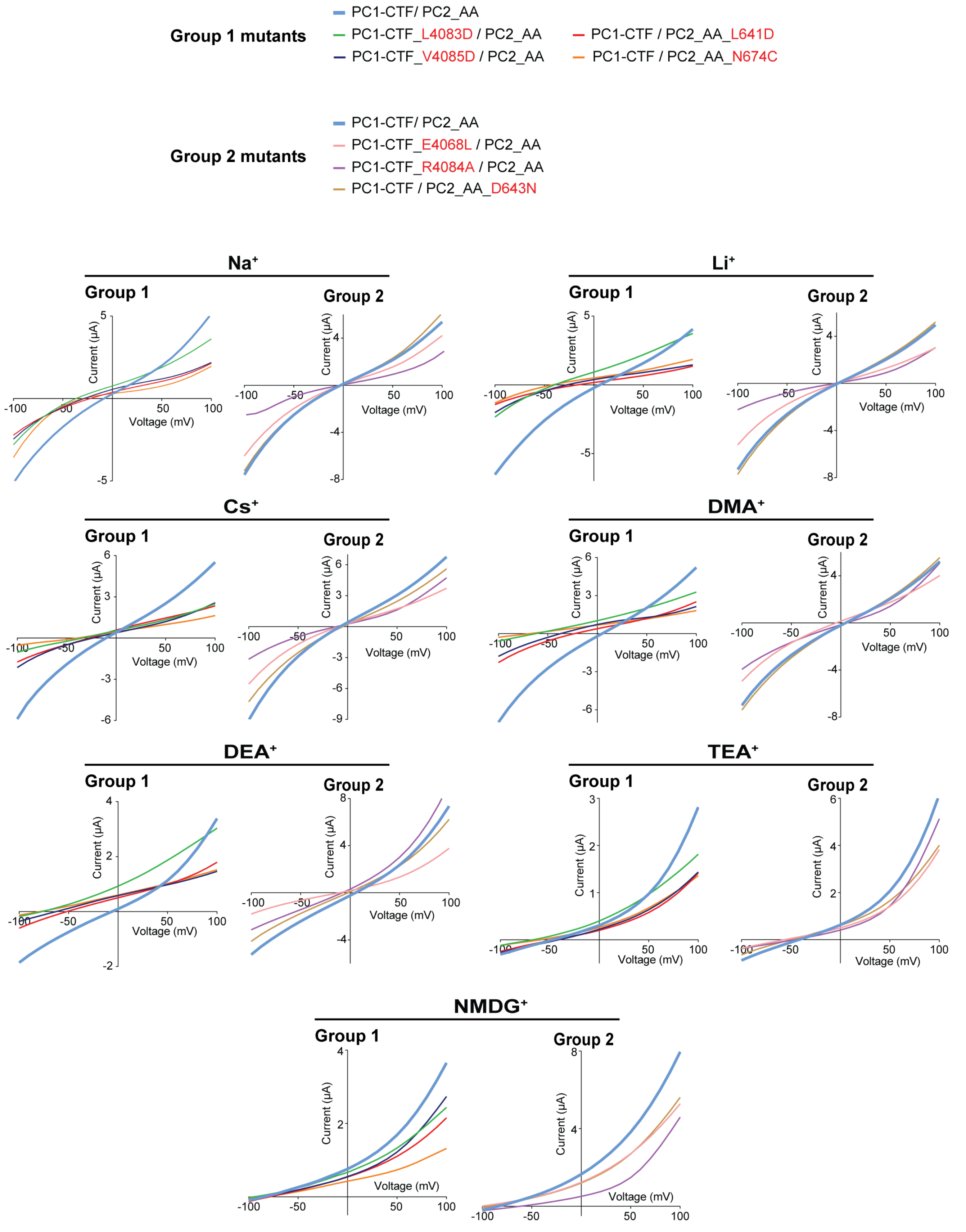

Figure EV5.

 © 2019 The Authors
