# Supplementary figures and images for: The ion channel function of polycystin‐1 in the polycystin‐1/polycystin‐2 complex
Source: EMBO Rep. 2019 Aug 22;20(11):e48336. doi: 10.15252/embr.201948336 (PMC6832002; doi:10.15252/embr.201948336)

**Fig. S4**

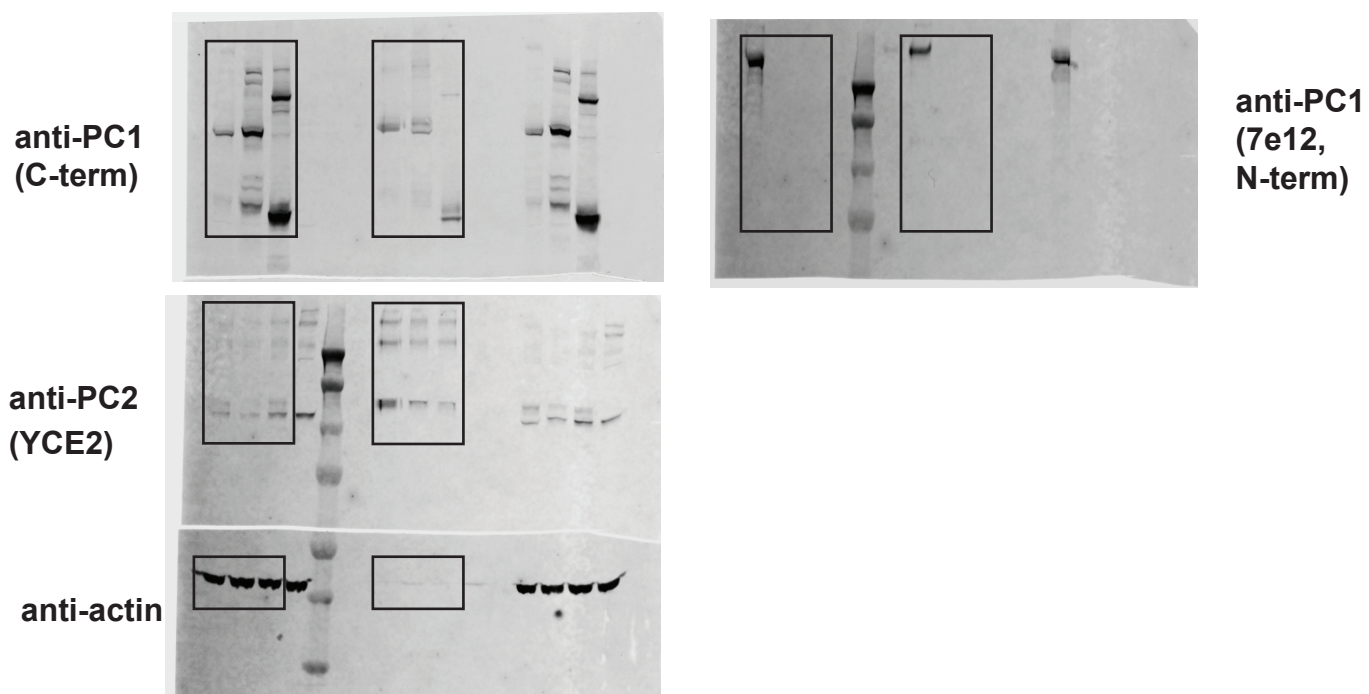

Supplement: Supplementary file 3 — Source Data for Expanded View and Appendix [file EMBR-20-e48336-s010.zip › EMBOR-2019-48336-T_SourceDataForFigureS4.pdf]

**Fig. 1B**

anti-PC1

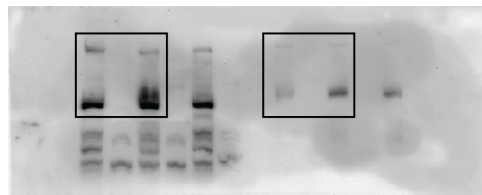

anti-HA

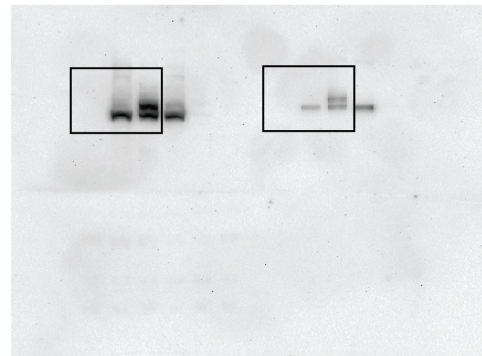

anti-actin

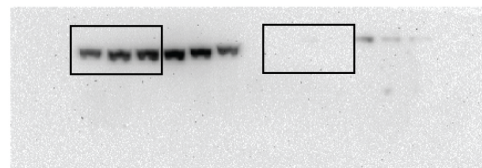

**Fig. 1C**

anti-PC1

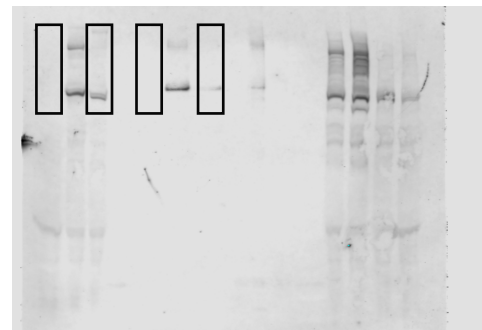

anti-HA

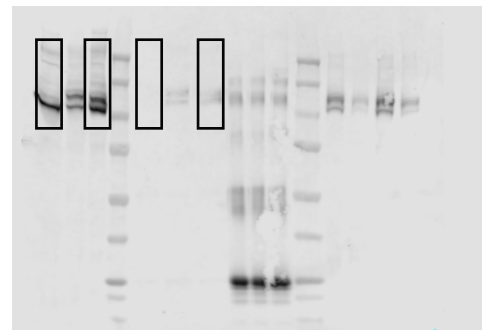

Supplement: Supplementary file 5 — Source Data for Figure 1 [file EMBR-20-e48336-s003.zip › Source_Data_for_Fig1/Source_Data_for_Fig1.pdf]

**Fig. 3H**

**anti-PC1**

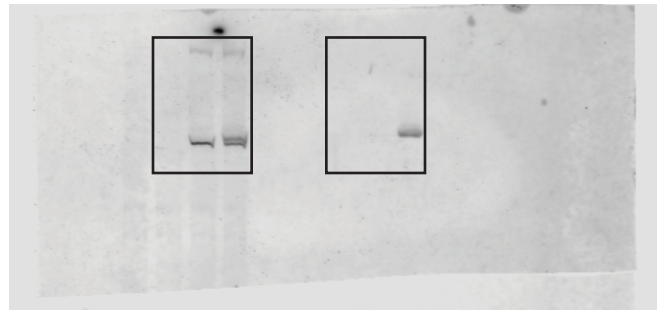

**anti-HA**

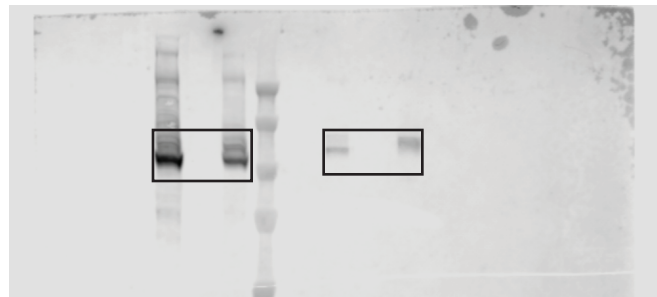

**anti-actin**

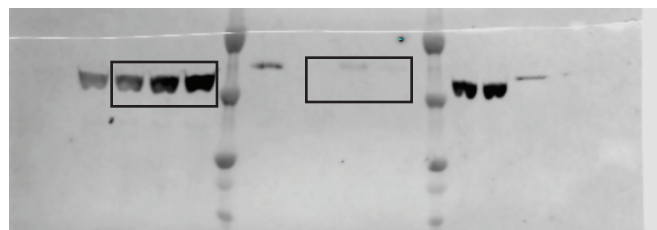

Supplement: Supplementary file 7 — Source Data for Figure 3 [file EMBR-20-e48336-s005.zip › Source_Data_for_Fig3/Source_Data_for_Fig3.pdf]

**Fig. 4E**

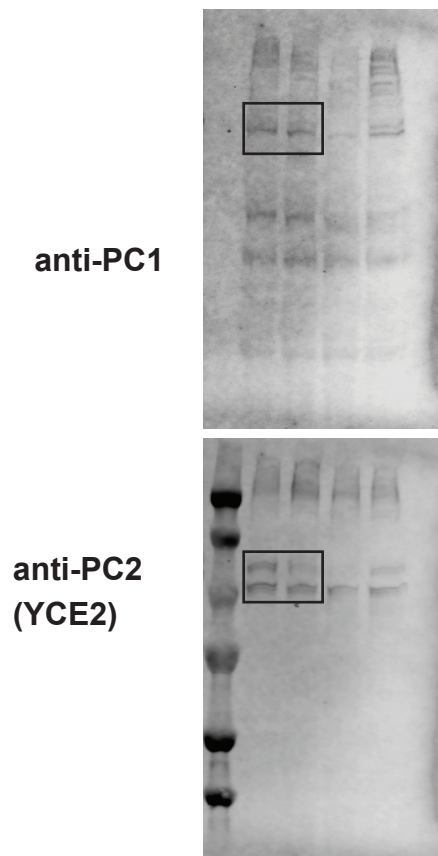

**Fig. 4I**

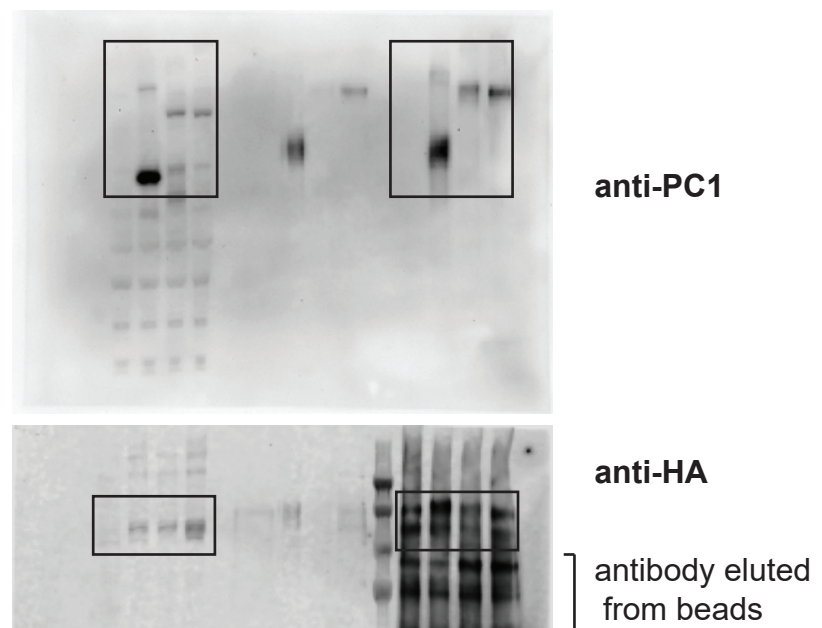

**Fig. 4J**

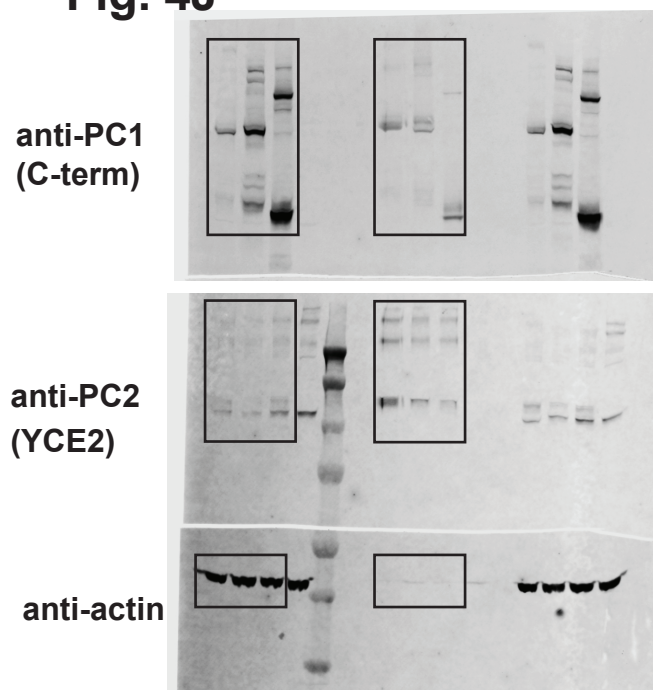

Supplement: Supplementary file 8 — Source Data for Figure 4 [file EMBR-20-e48336-s006.zip › Source_Data_for_Fig4/Source_Data_for_Fig4.pdf]

**Fig. 7D**

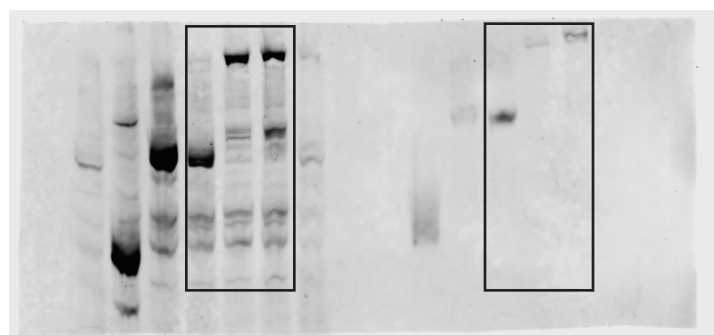

**anti-PC1**

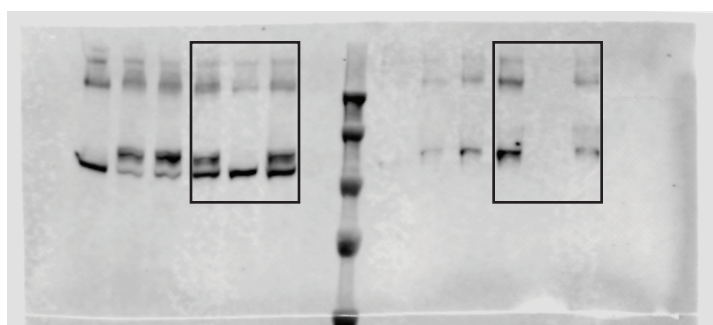

**anti-HA**

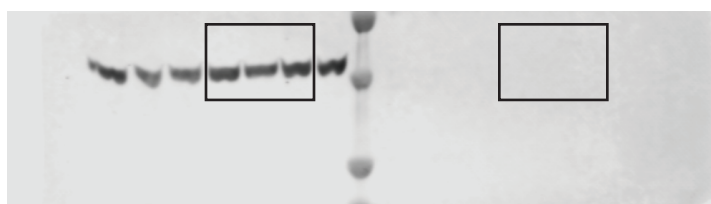

**anti-actin**

Supplement: Supplementary file 11 — Source Data for Figure 7 [file EMBR-20-e48336-s009.zip › Source_Data_for_Fig7/Source_Data_for_Fig7.pdf]
